# Supplementary material for: RAC1b Collaborates with TAp73α-SMAD4 Signaling to Induce Biglycan Expression and Inhibit Basal and TGF-β-Driven Cell Motility in Human Pancreatic Cancer
Source: Biomedicines. 2024 Jan 16;12(1):199. doi: 10.3390/biomedicines12010199 (PMC10813112; doi:10.3390/biomedicines12010199)
Supplement: Supplementary file 1 [file biomedicines-12-00199-s001.zip › biomedicines-2758355-supplementary.pdf]

Figure S1 (uncropped blots from Figure 3A)

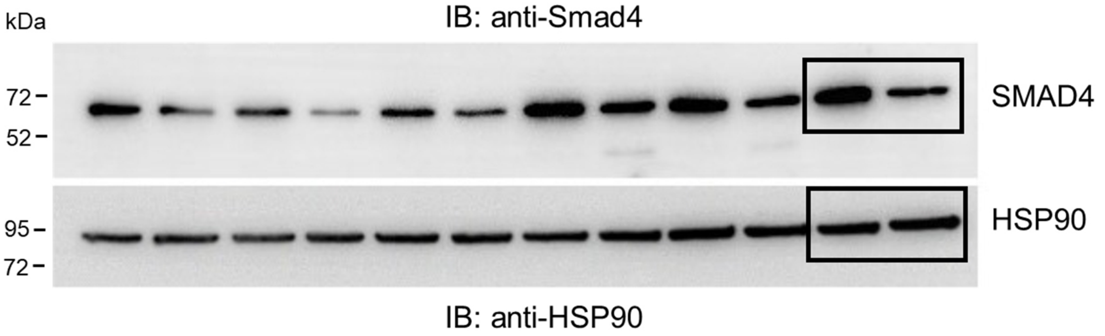

Figure S2 (uncropped blots from Figure 4A)

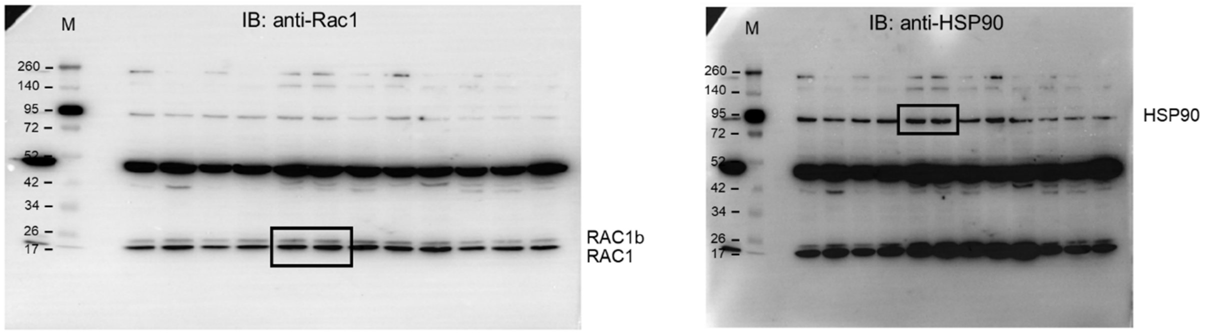

Figure S3 (uncropped blots from Figure 6D)

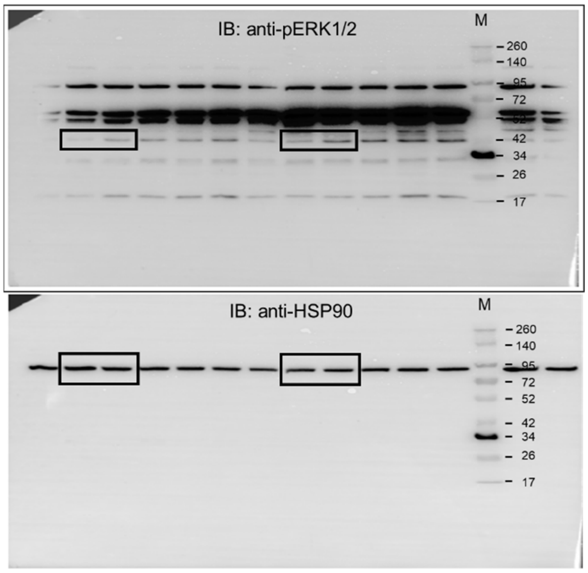

The boxed bands indicate those in the main figure. Figure legend: M = molecular weight marker (SM1841, Fermentas/Thermo Fisher Scientific)

Figure S4: qRT-PCR-based verification of ectopic overexpression of BGN in PANC-1 cells

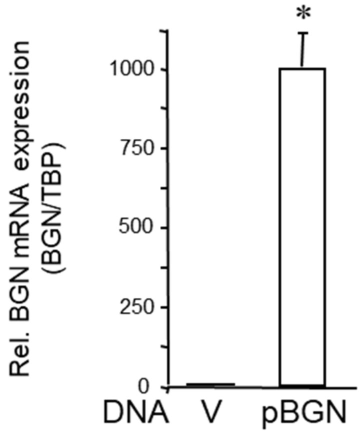

Figure S4. PANC-1 cells (2.5x10^{5}) grown in 12-well plates were transiently transfected for 4 h with Lipofectamin 2000 and 1.6 µg each of empty pcDNA3.1 vector (V) or a human BGN cDNA in pcDNA3.1 (pBGN). Forty-eight h after the end of transfection, cells were lysed and analyzed by qRT-PCR for expression of BGN and the housekeeping gene TBP. Data represent the normalized mean ± SD (n = 3). The asterisk (*) indicates a significant difference relative to the V control. The primer sequences for BGN and TBP can be found in [16].

Figure S5 (uncropped blots from Figure 7A,B)

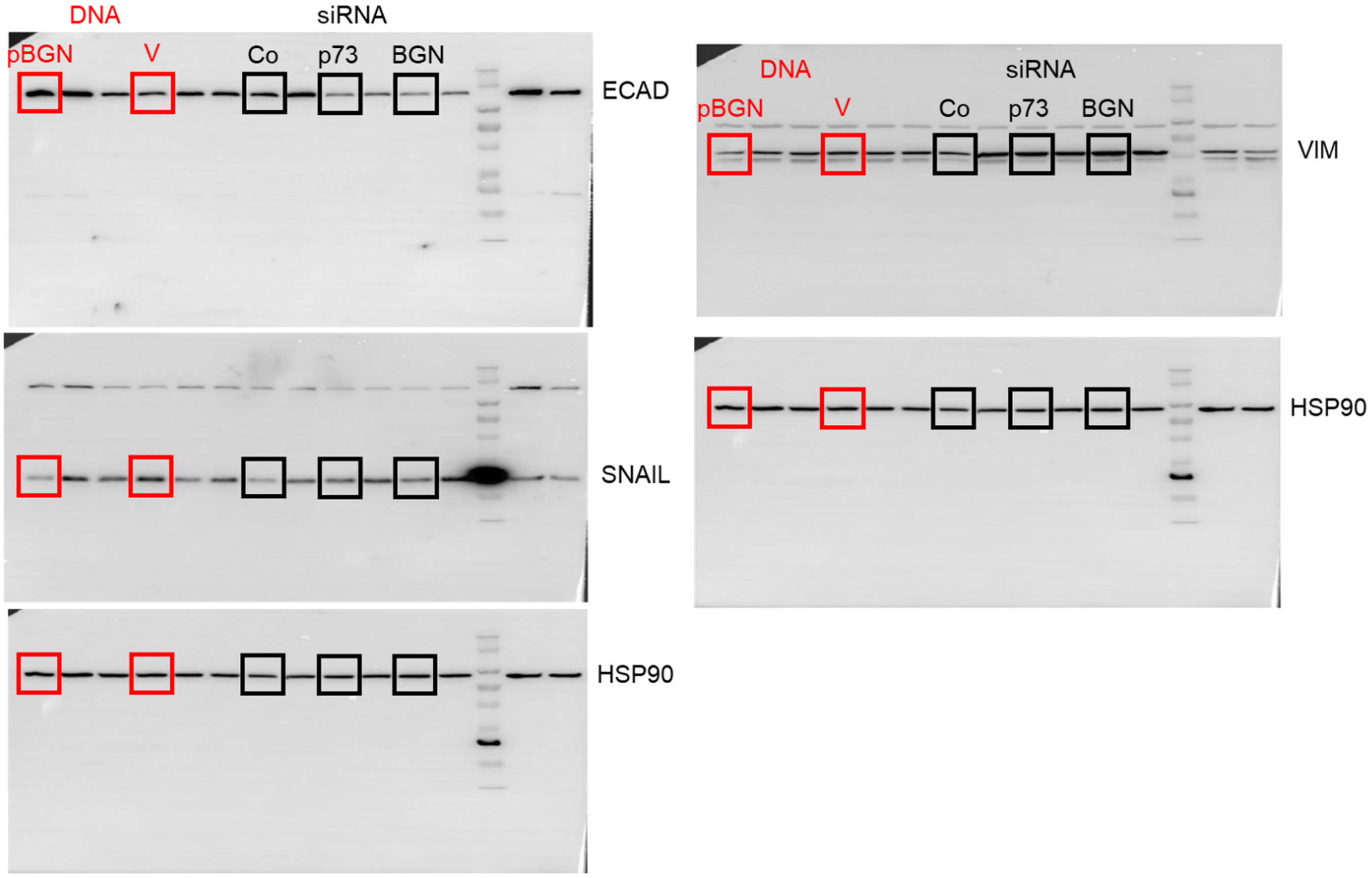

The boxed bands indicate those in the main figure. Figure legend: M = molecular weight marker (SM1841, Fermentas/Thermo Fisher Scientific)

Figure S6 (uncropped blots from Figure 7D)

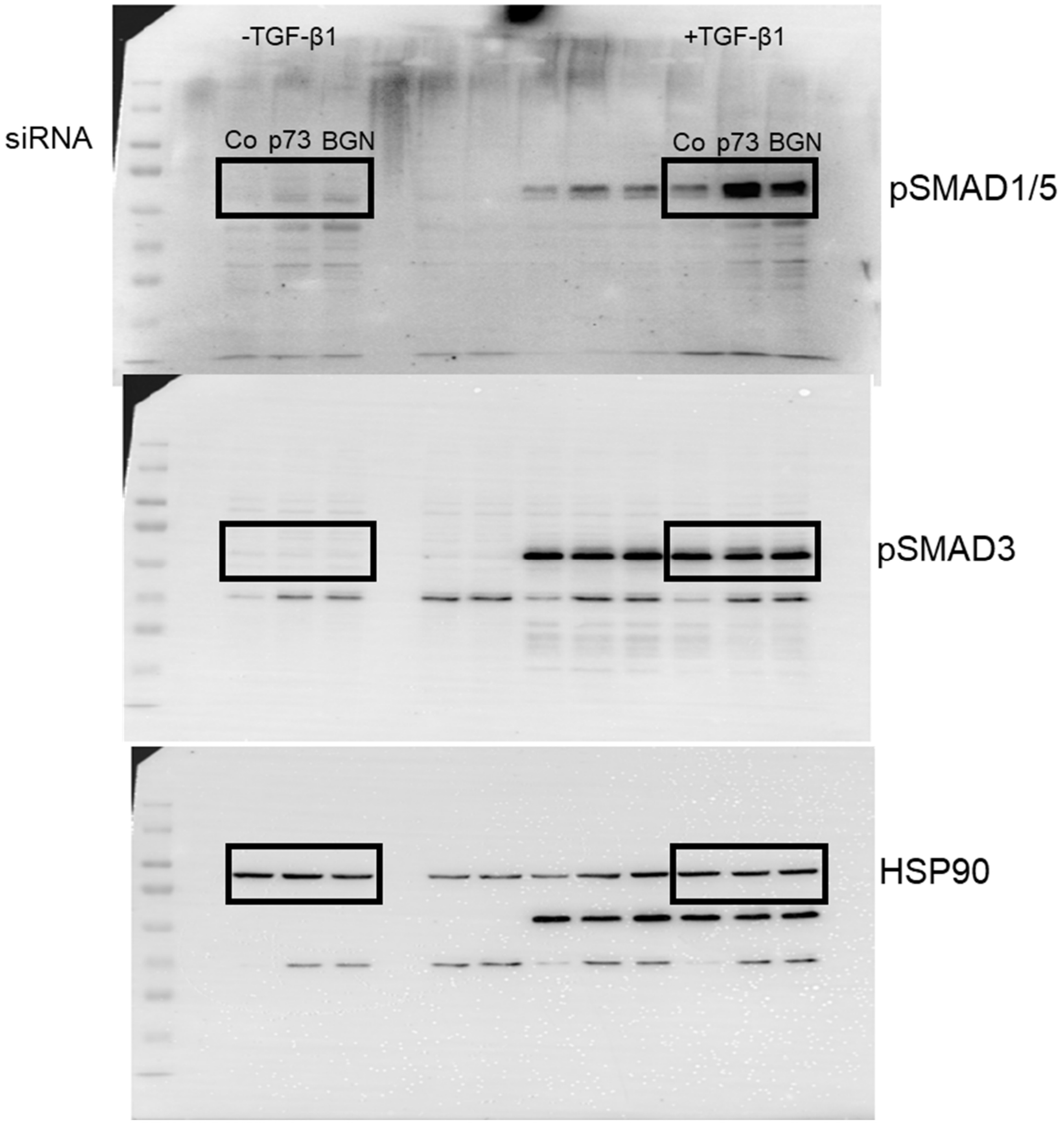

The boxed bands indicate those in the main figure. Figure legend: M = molecular weight marker (SM1841, Fermentas/Thermo Fisher Scientific)
